# Analyzing Body Movements within the Laban Effort Framework Using a Single Accelerometer

**DOI:** 10.3390/s140305725

**Published:** 2014-03-21

**Authors:** Basel Kikhia, Miguel Gomez, Lara Lorna Jiménez, Josef Hallberg, Niklas Karvonen, Kåre Synnes

**Affiliations:** Department of Computer Science, Electrical and Space Engineering, Luleå University of Technology, Luleå 971 87, Sweden; E-Mails: miguelgomezsimon@gmail.com (M.G.); laraljj@gmail.com (L.L.J.); Josef.Hallberg@ltu.se (J.H.); Niklas.Karvonen@ltu.se (N.K.); Kare.Synnes@ltu.se (K.S.)

**Keywords:** Laban movement analysis, effort category, accelerometers, machine learning, body movements, accelerometers placement

## Abstract

This article presents a study on analyzing body movements by using a single accelerometer sensor. The investigated categories of body movements belong to the Laban Effort Framework: Strong—Light, Free—Bound and Sudden—Sustained. All body movements were represented by a set of activities used for data collection. The calculated accuracy of detecting the body movements was based on collecting data from a single wireless tri-axial accelerometer sensor. Ten healthy subjects collected data from three body locations (chest, wrist and thigh) simultaneously in order to analyze the locations comparatively. The data was then processed and analyzed using Machine Learning techniques. The wrist placement was found to be the best single location to record data for detecting Strong—Light body movements using the Random Forest classifier. The wrist placement was also the best location for classifying Bound—Free body movements using the SVM classifier. However, the data collected from the chest placement yielded the best results for detecting Sudden—Sustained body movements using the Random Forest classifier. The study shows that the choice of the accelerometer placement should depend on the targeted type of movement. In addition, the choice of the classifier when processing data should also depend on the chosen location and the target movement.

## 1. Introduction

Analyzing Body Movements is receiving an increasing amount of attention from context-awareness researchers. This attention is motivated by the wide range of applications that rely on the analysis of human motion. For instance, analysis of dancer/athletic performance, medical diagnosis [1], and recognizing emotions based on the movements of the body [2]. The movements of the body usually form a synchronized pattern when performing activities. Those patterns can be analyzed and described in the framework of Laban Movement Analysis (LMA). LMA is a method for describing and interpreting all varieties of human movements. It provides a rich overview of movement possibilities, and it is considered as “a formal language for movement description” [3]. LMA is divided into four categories: Body (total-body connectivity), Effort (Energetic dynamics), Shape, and Space [4]. This work focuses on analyzing body movements with regard to the Effort category, which is also divided into four subcategories: Strong—Light, Sudden—Sustained, Bound—Free and Direct—Indirect [5].

It is demonstrated in [6] that even an expert cannot categorically determine whether a movement is Strong, Light, Sudden, Sustained, Bound, Free, Direct or Indirect. This classification may even vary from one expert to another. It is therefore important to define each type of movement beforehand, so it is possible to choose the right activities that represent each movement when collecting data. The authors have defined the types of movement within this study as follows:

- Strong: A movement is considered to be Strong when a person needs to make a considerable effort to perform an activity.
- Light: A Light movement is such that a person could perform the activity effortlessly.
- Free: A movement is considered to be Free when it is characterized by open postures where the extremities of the body, mainly upper body limbs, are kept mostly away from the body.
- Bound: A Bound movement is a controlled movement performed with the extremities close to the body.
- Sudden: A Sudden movement is a swift movement that does not follow any particular pattern. It generates a change in velocity, that is, a spontaneous acceleration.
- Sustained: A Sustained movement is a continuous movement that follows a specific pattern where the velocity is maintained.
- Direct: A movement is considered to be Direct when the route a person follows over a certain period of time is on average a straight path.
- Indirect: A movement is considered to be Indirect when a person follows, over a certain period of time, an oblique route.

Naturally, we perform all these movements during our daily activities. For instance, most persons walk effortlessly, and the required movement is considered to be Light, but carrying heavy objects while walking will increase the activity's Effort level, and the movement would be considered as Strong. To recognize activities of daily living, many researchers have used wearable sensors for the task of human activity recognition. In particular, machine-learning techniques have been utilized for the purpose of using accelerometers to detect daily activities such as walking, running, sitting and lying [7–10]. The small size of accelerometers and their low power consumption make them well suited to wearable applications [11]. However, the purposes of the classified data from movement analysis and activity recognition are different. The primary purpose of movement analysis is to determine the movement effort, either for the use on its own or to be combined with other contexts to clarify the current situation. For instance, differentiating between strong exercise and strong emotions when the movement classification is coupled with a galvanic skin response sensor that measures the subject's stress [12]. On the other hand, the primary purpose of classical activity recognition classification is to gain direct insight into the specific type of activity.

**Figure 1.**
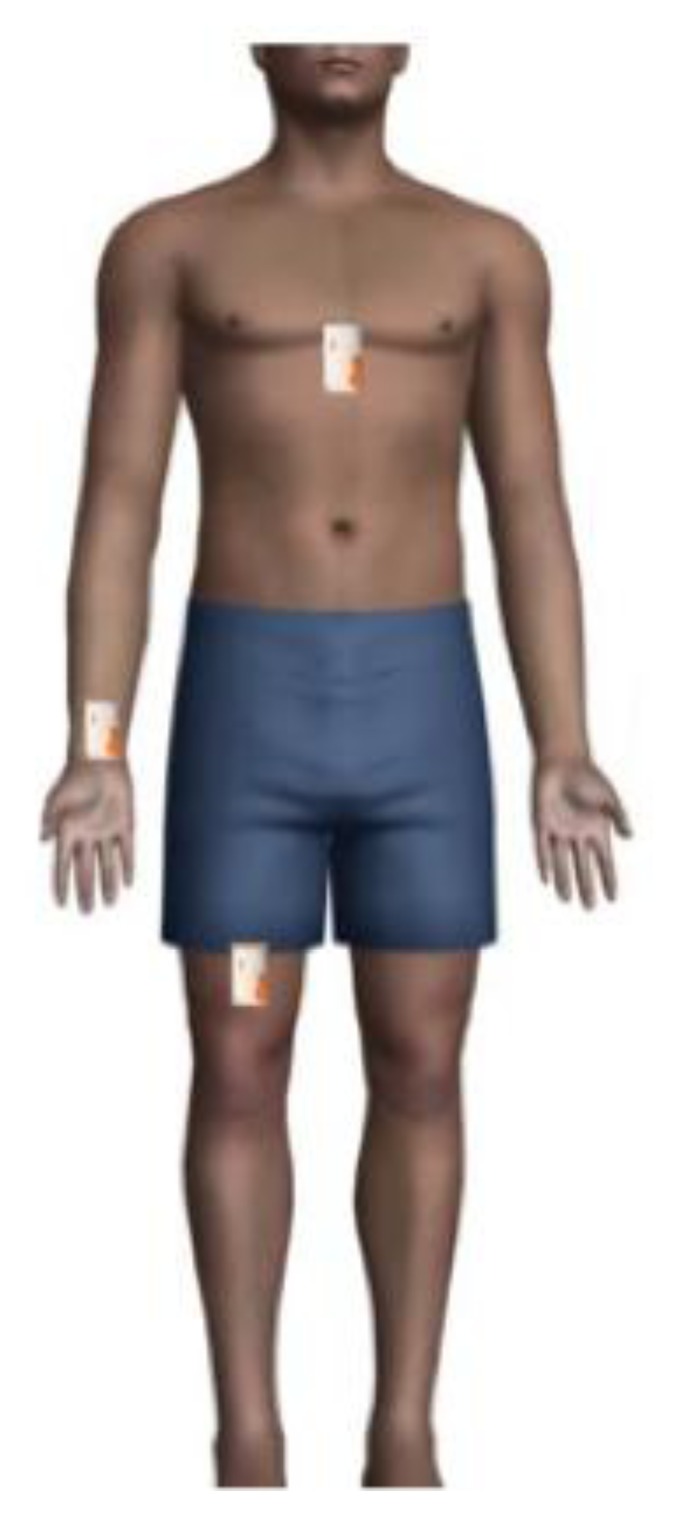
Selected placement locations for the accelerometers (chest, wrist and thigh). The wrist and thigh sensors are placed at the dominant side of the body.

In this article, the authors present an experiment to categorize the body movements of the subject using wireless tri-axial accelerometers placed at the chest, wrist and thigh. Those locations have shown positive results for detecting activities of daily living in [8,13–15]. Figure 1 illustrates the placement of the accelerometers at the chosen locations.

Having multiple sensors will increase the complexity of the monitoring system and make it more cumbersome for the subject [16]. The authors investigate the best location between chest, wrist and thigh, to place a single accelerometer for the purpose of detecting each type of movement. The aim of the work is to answer the following research questions:

1. What level of accuracy can be achieved in detecting body movements within the Effort category using a single accelerometer?
2. Which are the best machine-learning techniques and the best placement for an accelerometer to accurately classify each type of movement within the Effort category?

The results of the presented work in this article will give an indication of how to estimate the physical level of the body movements. This estimation can be employed in different applications. For instance, physiotherapists can get an estimation of the body movements' level of their patients throughout the therapy, and dance teachers can get an estimation of the body movements' level of their students while dancing. Note that this work does not cover the classification of the Direct and Indirect elements within the Effort category. Those elements generally require a non-accelerometer external subsystem in order to capture them, such as GPS subsystem.

The rest of this paper is organized as follows: Section 2 presents state-of-the-art related work. Section 3 discusses the data collection, which includes the chosen activities to represent each type of movement, and the data collection process by the participants of the study. Section 4 presents how the collected data has been processed, this includes the features that have been extracted and selected, and the classification models that have been built and tested. Section 5 shows the obtained results, and section 6 discusses the research questions. Finally, Section 7 concludes the paper and presents future work.

## 2. Related Work

Some previous studies classified movements performed by subjects within the Laban Movement Analysis framework (LMA). For example, Fagerberg *et al.* [17] classified the body movements within LMA to find the connections between the mental state of the subject and the movements being performed. The traditional methodology was employed for this purpose based on the observation of the movements by either a Laban expert [6] or movement experts, such as choreographers or expert dancers [18]. Some researchers have tried to capture the movements of individuals using the human interaction with a system. Mentis *et al.* [6] used video data captured by a Kinect camera to analyze the movement qualities. The movement qualities were calculated based on acceleration, pathways, velocity, levels and relationship of limbs to the body. The movements captured from the subject were contrasted with the opinion of various Laban experts for the purpose of seeing whether the system was able to recognize the movements or not. The study provided indications of how movement qualities can be detected using a static video camera, and how these qualities can be integrated into the design of interactive systems. However, this system showed weakness when the recognition of the movements is conducted in a real world situation, since the system is not portable and it requires a controlled environment. Another study presented by Foroud *et al.* [19] that focused on analyzing the movements of rats instead of human beings. The movements created by rats when interacting with each other have been collected and stored in videotape. Using the traditional methodology, the videotape was analyzed and the movements were classified within the LMA Effort factors.

Godfrey *et al.* [16] presented a review about measuring the human movements by the use of accelerometers. The reason of focusing on accelerometers was the low cost, weight and power consumption. Other techniques were discussed for collecting the movements such as the use of diaries, questionnaires or observation. These techniques were rejected because they present disadvantages when a continuous analysis of the movements is required. The review presented a compromise between the number of accelerometers and the data obtained from them. The authors in [16] affirmed that a smaller number of accelerometers makes the monitoring of movements less complex, whereas the amount of information is reduced. The results also showed that the use of accelerometers is a good way to collect movements. In addition, the authors concluded that accelerometry is a non-intrusive mean to access ambulatory movements, postures, postural transitions and intensity of movements. Another study was done by Veltink *et al.* [20] to distinguish between postures and movements of the body. The authors used two uni-axial accelerometers placed at the trunk and the upper legs for the detection purpose. The study showed that it is possible to distinguish between postures and movements by simply checking the sensors signal. If the signal is not changing over time, it can be assumed that the body segment is not moving. However, the discrimination between various movements was not analyzed.

Most of the previous works in this field have focused on the use of accelerometers for the purpose of recognizing daily activities. The method that was generally implemented was to collect data using accelerometers and then use machine-learning techniques to process it. For instance, activity recognition has been done in [8,10,13–15]. Some of these studies use tri-axial accelerometers [8,13–15] placed at different parts of the body such as chest, wrist, thigh, waist and ankle, while others use bi-axial accelerometers [10]. Olguin and Pentland [21] used accelerometers attached to the chest, hip and wrist to detect activities of daily living and the accuracy rate was up to 92%. Another study done by Ravi *et al.* [15] used a single accelerometer attached to the waist to detect a range of activities and the accuracy of detection was up to 64%. One major topic that is discussed in related works is the optimal placements when using accelerometers. Cleland *et al.* [8] investigated the use of five tri-axial accelerometers placed at the chest, wrist, lower back, hip, thigh and foot to detect seven activities of daily living. Data collected from the hip yielded the best results for detecting activities using a Support Vector Machine classifier. Bao and Intille [10] used five biaxial accelerometers worn on the user's right hip, dominant wrist, non-dominant upper arm, dominant ankle and non-dominant thigh when collecting data. Their work suggested that using an accelerometer at the dominant wrist and thigh may be able to detect common everyday activities. Different sensor locations have been studied by for example Cleland *et al.* [8], Gjoreski *et al.* [13] and Atallah *et al.* [14]. These studies analyzed the different locations and their performances as a data source for detecting a set of everyday activities.

The authors in this article investigate the possibility of classifying body movements within the Laban Effort category using a single accelerometer. To the best of the authors' knowledge, this was not investigated before. In addition, the presented work discusses the sensors' placement for the purpose of detecting body movements instead of activities, which complements and expands previous works in this field.

## 3. Data Collection

### 3.1. Definition of Activities

It may be sufficient to detect the intensity of the performed activities in order to indicate the type of movement included. A set of activities is defined for each type of movement within the Effort category. The subject is expected to perform some actions during each activity and those actions will give an indication about the body movements. The total number of chosen activities is 23. Some of the activities were repeated for different types of movement with slight variations in the way the activity was performed.

#### 3.1.1. Sustained, Light, Bound and Strong Activities

For the first three elements (Sustained, Light and Bound), the subject was asked to perform seven activities, namely walking, running, stairs up, stairs down, sitting, lying and standing. Each activity was performed for one minute. The element Strong contains a variation of some of the mentioned activities. For Strong movements, the subject was asked to carry some heavy objects in a box and also in a backpack while performing walking, running, stairs up and stairs down. The purpose is to increase the activity's Effort level so the subject would feel it as a “strong activity”. In addition, the subject was asked to perform a cycling activity with first gear for Light, second gear for Sustained and Bound, and third gear with heavy items for Strong.

#### 3.1.2. Sudden Activities

Each activity performed in this element should be comprised of a series of sudden movements. Each activity was performed for one minute. The defined activities are the following:

- Finding a cell phone: A cell phone was hidden in a room. The phone was called and the subject had to find it. When doing this activity the subjects perform abrupt body movements, particularly involving the upper body, in their haste to find the cell phone.
- Putting shoes on and taking them off: The subject produces swift and non-patterned movements, especially hand movements.
- Getting dressed: the subject was asked to put on a winter cap, a pair of gloves, a scarf and a coat, and then take them off. The movements a subject performs while putting on each item are fast during a short period of time.
- Simon says, a kid's game: In this game the researcher stated an activity (*i.e.*, walking/running) and the subject has to perform it. The intent is to alternate rapidly between the activities performed and therefore, generating sudden movements. These activities were: Walking, running, jumping, hopping right leg, hopping left leg and stopping.
- Cleaning: In an open room, some trash was placed on the floor and on a table so the subject would have to sweep the floor and use a cloth to clean the table. Some notes were written on a whiteboard so that with the help of an eraser, it had to be cleaned by the subject. The subjects produced sudden movements due to the nature of the activity of cleaning.
- Making a sandwich: a few ingredients were scattered around a room to create sudden and non-patterned movements. The subjects were asked to make a sandwich by choosing the ingredients they saw fit.

#### 3.1.3. Free Activities

**Figure 2.**
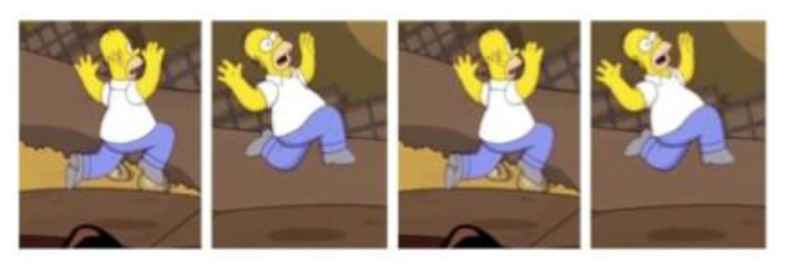
Homer in the Land of Chocolate.

**Figure 3.**
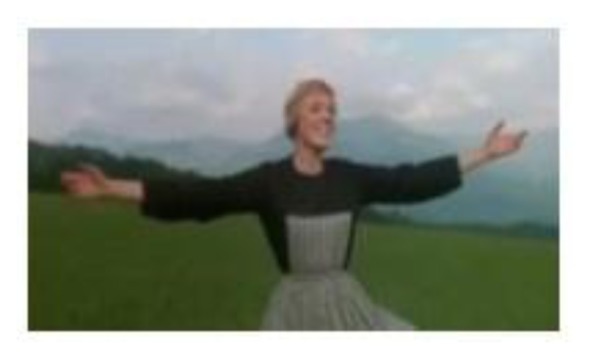
The Sound of Music.

Three activities were defined for this element. The activities were chosen because they generate a series of free movements when performing them:

- Dancing: For this activity each subject was granted a private space in an effort to not make the individual feel self-conscious and therefore negatively impacting the data collection of this activity. Dancing has twice the samples the rest of activities do (two minutes), in order to cover some of the sample shortage in this category.
- Running like “Homer in Land of Chocolate”: This combination of activities was extracted from “The Simpsons” TV show where one of the main characters was generating free movements throughout the activity. It is an activity in between running and jumping as it is shown in Figure 2. This activity was performed for one minute.
- Walking like “Sound of Music”: This activity is based on the beginning of the classic film “The Sound of Music”. The idea is to collect free movement as shown in Figure 3. This activity was performed for one minute.

### 3.2. Collecting Data

Ten healthy subjects participated in the data collection. The participants were students at Luleå University of Technology. Information about the subjects is presented in Table 1 below.

The subjects were equipped with three Shimmer wireless sensors (Shimmer 2R, Realtime Technologies, Dublin, Ireland) placed at the chest, thigh and wrist, as shown in Figure 1. The Shimmer sensors were fixed to the body using elasticized strapping and holsters. This is a common method of attachment in activity recognition studies [11]. The thigh and wrist sensors were placed depending on whether the subject was left handed or right handed (in the dominant side of the body), and also aligned to the chest sensor. The subjects were also equipped with a smartphone using a sport's armband on the non-dominant arm, and with a stopwatch to notify them when to stop performing the activity. The shimmer sensor allows ranges of acceleration of ±1.5 g and ±6 g. It is stated in [22] that ±2 g was insufficient to determine vigorous exercise, while other studies have shown that the use of ±6 g acceleration range can be sufficient [23,24]. The range of acceleration was therefore set to ±6 g. The sampling rate of the Shimmer sensor was set to 10 Hz, which is considered to be sufficient for detecting daily activities from accelerometer data [14,15].

A video of 3 min was prepared beforehand to demonstrate the activities that the subject had to perform. The focus of the video was on the free movements as it is not common to perform such movements on a daily basis. The video was played to each subject separately, and then the subject was asked to perform the 23 activities of one minute each (except for the dancing activity, which is performed for 2 min). The data was transmitted via Bluetooth to the mobile device that the subject had attached to the arm, where it was saved for later offline analysis. The raw acceleration data was labeled after each activity based on the performed movement. The subjects were asked from time to time to give feedback on the position of the sensors on their bodies. In addition, the position of the sensors was checked after each activity, and a correction was made when needed. The list of the performed activities by each subject is summarized and shown in Table 2 below. The number of samples refers to the ones collected from each location (chest, wrist and thigh).

## 4. Data Processing

### 4.1. Feature Extraction

As shown in Table 2 above, the total number of acceleration samples was around 23,400 per subject from each location. Having 10 subjects produces around 234,000 samples for all types of movement from chest, wrist and thigh. Features were extracted from the raw acceleration data using a window size of 128 samples with 64 samples overlapping between consecutive windows. This represents 12.8 s of data per window. A 50% window overlap has been deemed sufficient to compute the features [10], and the window size was selected in a trade-off between fast computation of the data and the ability to accurately determine cyclic movements [7,25–27]. Eighteen features were extracted from each window, giving a total of 34 attributes. Table 3 below presents all the extracted features.

The extracted features are a combination of time-domain (features 1 to 14) and frequency-domain (features 15 to 18). Time-domain features grant the possibility of differentiating dynamic movements from static ones [13,27]. Frequency-domain features are essential for identifying patterns within acceleration data, which aids in discriminating vigorous from moderate movements [10,28].

Features 1–14 are standard statistical metrics. For feature 15, it has been concluded in [27] that FFT components have a greater assessing ability than other features. The authors in [27] analyzed which number of FFT components maximizes the classification accuracy attained, where the first five FFT components were used. Feature 16 (Spectral energy) is calculated as the sum of the squared FFT components within the chosen window. Afterwards, the result obtained is normalized employing the window length. This feature is particularly relevant when assessing the energy expenditure of a subject while moving [8]. For the last feature (feature 18), it is well known that when a subject performs an activity, a certain range of frequencies is generated [26]. Making use of the main frequency feature may prove useful in determining various types of movements.

### 4.2. Feature Selection

To avoid redundant or irrelevant features, feature selection was done over the set of the extracted features. A filter method (InfoGain) was used to obtain the best set of features that provides the highest accuracy and minimizes overfitting [29]. To do that, the collected data was first organized based on the location of the accelerometer and the subcategory within LMA. For instance, acceleration data collected from the chest sensor labeled as Strong or Light (for all 10 subjects) was grouped together in 1 file. This was repeated for each location (chest, wrist, thigh) in combination with each subcategory (Strong—Light, Sudden—Sustained, Bound—Free). This procedure created nine files that represent (location-subcategory).

InfoGain method creates a ranking of the most relevant features based on the information obtained with respect to the class evaluated [30]. For each combination (location-subcategory), the 34 attributes were ranked using the InfoGain method. The features were then divided into different subsets recursively based on the previous raking using the Bisection algorithm [31]. Those subsets were tested later using the classification models to find the subset that gives the best accuracy. All attributes were used if there was no subset that gave better results. Finding a subset with the most relevant features increases the accuracy and decreases the computation time.

### 4.3. Classification Models

Eight machine-learning classifiers have been evaluated based on their classification accuracy: Naïve Bayes [13,15], C4.5 [11,15,32], Logistic Regression [32], Support Vector Machine (SVM) [22,33], K-Nearest Neighbour [14,27,33], Random Forest [33,34], Boosting [15,33] and Bagging [33,34]. These classifiers have been implemented in previous works, and they have shown good results that vary based on the nature of the study. The WEKA data mining software (Version 3.6.7, University of Waikato, Hamilton, New Zealand) has been used to build the classifiers. WEKA supports several standard data mining tasks, more specifically, data pre-processing, clustering, classification, regression, visualization, and feature selection [35].

The extracted features were tested with the chosen classifiers to select the best features for each combination (location-subcategory). After selecting the best set of features for each combination, Leave-one-subject-out cross validation (LOOCV) [10] was used to train each classifier on nine subjects and uses the subject excluded for testing. This procedure was repeated ten times (one per subject) excluding a different subject each time. The average of the results was calculated to give the accuracy of the classifier in detecting the right movement. Leave-one-subject-out cross validation guaranties that there will be no overlapping data between the training set and the test set, which will generally give more realistic results.

## 5. Results

The eight classifiers were tested using Leave-one-subject-out cross validation (LOOCV) for each subcategory within the Effort category. The collected data by the 10 subjects was used for training and testing each classifier. The level of accuracy was different among various classification methods and locations. Table 4 presents the results for (Strong—Light), Table 5 for (Sudden—Sustained) and Table 6 for (Bound—Free). Each row in each table represents the level of accuracy obtained when using a single accelerometer placed at a specific location on the body.

The best classifier for each location, in terms of accuracy, is marked with bold text in the previous tables. In addition to the classification accuracy, the F-measure value was calculated. The F-measure was used as a performance index to evaluate how reliable these results are. It is a combined metric that combines precision and recall as presented in Equation (1):

(1) $$F-\mathrm{measure}=2\times \frac{\mathrm{precision}\times \mathrm{recall}}{\mathrm{precision}+\mathrm{recall}}$$

Precision is the proportion of instances which truly have class x among all those which were classified as class x. For instance, a precision 0.85 means that 85% of the returned documents were relevant. Recall is the ratio of relevant documents found in the search result to the total of all relevant documents. A higher value of recall indicates that relevant documents are returned more quickly [30]. Ideally the best classifier would give an accuracy of 100% and an F- measure value of 1. Table 7 below presents the best classifiers based on the previous tables, including the F-measure values.

In light of these results, Table 8 below summarizes the best placement of an accelerometer for detecting each subcategory within the Effort category.

Based on Table 7 above, it is also possible to calculate the average accuracy of each location for detecting all subcategories within the Effort category. Table 9 below shows the average accuracy that can be obtained from a single accelerometer for detecting all subcategories.

## 6. Discussion

This section discusses the results of the efforts with respect to the research questions. Firstly, it is important to note that even though the aim of LMA is to standardize the classification of human movement, there is room for interpretation within this framework. Differentiating between Strong—Light, Sudden—Sustained, and Bound—Free movements is a matter of subjective opinion. While the baseline may differ between individuals, the difference between movement types should be easily separable. Making the baseline configurable should therefore reveal quantifiable thresholds that would allow differentiating between movement types. The presented results in this article discuss the analysis of human movement based on the definition of movements, listed in Section 1, and the definition of activities, listed in Section 3.1. These results might differ if an expert has a different opinion of what each type of movement could be. In addition, movement-based recognition differs from the common activity-based recognition systems. There are many examples of applications that can benefit of using LMA instead of using activity-based recognition systems. While an activity-based system focuses on the exact activity performed by the subject, LMA can give an overview of the subject's state and situation by analyzing the movements of the body. For instance, analyzing body movements of patients with physical injuries to help doctors follow the progress of the therapy.

The introduction section presented two research questions that have been discussed and addressed in this article. The first addressed question is: “*What level of accuracy can be achieved in detecting body movements within the Effort category using a single accelerometer?*”

As demonstrated in Tables 4, 5 and 6, the level of accuracy differs between classification methods and locations. In fact, the contents of these tables answer the first research question. For example, it is presented in Table 4 that the level of accuracy for detecting Strong – Light from the chest sensor range from 62.25% to 72.35% depending on the used classifier. This range is different when the aim is to detect other subcategories within the Effort category. As shown in Tables 5 and 6, the level of accuracy obtained from the chest sensor range from 78.15% to 87.51% for Sudden – Sustained and from 80.94% to 85.81% for Bound—Free, respectively. It is also noted that the worse/best classifier differs depending on the selected location and the target movement. The achieved level of accuracy leads to answer the second research question.

The second addressed research question in this article is: “*Which are the best machine-learning techniques and the best placement for an accelerometer to accurately classify each type of movement within the Effort category?*”

As it is presented in Table 7, the Random Forest classifier yielded the best results for the chest sensor in detecting all subcategories. The Random Forest was also the best classifier for the wrist sensor in detecting Strong—Light and Sudden—Sustained, while the best one for detecting Bound—Free movements is the SVM classifier. Even though SVM gave better results than Random Forest in detecting Bound—Free from the wrist data, the difference was small with a slight difference (87.31%, F-measure = 0.8694, for SVM, and 86.20%, F-measure = 0.8687, for Random Forest). It is therefore possible to use the Random Forest classifier with the chest and wrist sensors for detecting all subcategories within the Effort category. However, the best classifiers to classify data from the thigh sensor were Bagging forStrong—Light, K-Nearest for Sudden—Sustained and Boosting for Bound—Free. The choice of the classifier should therefore depend on the location of the accelerometer and the target movement. In addition, the classifiers' level of accuracy gives an indication of which is the best/worse suitable location to place an accelerometer for the purpose of detecting body movements.

For the best accelerometer placement between (chest, wrist and thigh), it is shown in Table 8 that the thigh location did not yield a best result for detecting any of the movements. The wrist was the best location for detecting Strong—Light and Bound—Free movements. However, the chest sensor gave the best results for detecting Sudden—Sustained. As a result, a single accelerometer should only be placed at the chest if the aim of the study is to detect Sudden—Sustained, and at the wrist for the other two subcategories. However, the wrist would be the best place if the aim were to detect each one of the subcategories simultaneously within the Effort category using a single accelerometer. This is shown in Table 9, as the wrist data gave the best average accuracy for detecting all subcategories simultaneously, with a value of 84.90%. In conclusion, the Random Forest classifier would be the best choice with an accelerometer placed at the wrist for the aim of detecting body movements within the Effort category.

The study shows that it is feasible to use a single accelerometer to analyze body movements within the Laban Effort Framework. This result is good because using a single accelerometer will reduce the burden of carrying multiple sensors in wearable systems, which makes such systems easier to use. For instance, a lifelogging system can utilize a single accelerometer placed at the wrist to analyze the body movements of the subject. This analysis can contribute into logging the life of the subject using a minimal set of equipment.

## 7. Conclusions and Future Work

This paper investigated the level of accuracy obtained when classifying body movements within the Effort category of Laban Movement Analysis using a single accelerometer. For this purpose, eight classifiers were tested on three separate body locations. Results show that in order to separately classify each subcategory (*i.e.*, Strong—Light) within the Effort category, both the best location and classifier vary from one subcategory to another. The wrist placement and the Random Forest classifier are the “best location” and “best classifier” respectively, since they are both the best at classifying body movements in two of the three subcategories. The results also demonstrate that the most successful classification is achieved for Sudden—Sustained, followed by Bound—Free and then Strong—Light.

The obtained results show that body movement can be classified within three subcategories of the Laban Movement Analysis Effort category with reasonable accuracy when using a single accelerometer. Future work should investigate this classification in an uncontrolled environment, where the subject would perform the movements naturally without instructions. Future work should also investigate the classification of body movements within the Direct—Indirect subcategory that has been left out of this study. For this purpose, an additional GPS subsystem should be employed. It is also important to focus on implementing the presented results within a lifelogging system that can take advantages of the analysis of body movements using a single accelerometer.

## Figures and Tables

**Table 1.** Subjects' information.

**Mean**		**Standard Deviation**
Height (m)	1.75	0.07
Weight (kg)	70.97	4.03
Age (years)	27.20	4.80
Right handed	1.00	0.00
Body Mass (kg/m^2)	23.11	1.95

Female	4	
Male	6	

**Table 2.** Activities performed by each subject.

**Laban Element**	**Activities**	**Time (min) Per Activity**	**Samples Per Activity**	**Total Samples Per Element from Each Location**
Sudden	Finding a cell phone—Putting shoes on and taking them off—Getting dressed—Simon says—Cleaning—Making a sandwich	1	≈600	≈3,600
Sustained	Walking—Running—Standing—Sitting—Stairs up—Stairs down—Lying—Cycling gear 2	1	≈600	≈4,800
Strong	Carrying Heavy Stuff and a backpack and performing: Walking—Running—Stairs up—Stairs down—Cycling gear 3	1	≈600	≈3,000
Light	Walking—Running—Standing—Sitting—Stairs up—Stairs down—Lying—Cycling gear 1	1	≈600	≈4,800
Free	Dancing—Running like “Homer in the Land of Chocolate”—Walking like “The Sound of Music”	1, except 2 for dancing	≈600, except ≈1200 for dancing	≈2,400
Bound	Walking—Running—Standing—Sitting—Stairs up—Stairs down—Lying—Cycling gear 2	1	≈600	≈4,800

**Total number of samples per subject for all 6 elements from each location**				**≈23,400**

**Table 3.** Features extracted from each window of raw acceleration data.

**Feature No.**	**Feature Description**
1	Acceleration Vector Magnitude (Length) value over 3 axes
2	Mean value for each axis (x, y, and z)
3	Average Mean value over 3 axes
4	Mean value over Length attribute
5	Root Mean Squared (RMS) value for each axis (x, y, and z)
6	Average RMS value over 3 axes
7	RMS value over Length attribute
8	Standard Deviation (STD) value for each axis (x, y, and z)
9	Average STD value over 3 axes
10	STD value over Length attribute
11	Skewness value for each axis (x, y, and z)
12	Average Skewness value over 3 axes
13	Kurtosis value for each axis (x, y, and z)
14	Average Kurtosis value over 3 axes
15	First 5 Fast Fourier Transform (FFT) value for each axis (x, y, and z)
16	Spectral Energy value for each axis (x, y, and z)
17	Average Spectral Energy value over 3 axes
18	Principal Frequency value for each axis (x, y, and z)

**Table 4.** Classification accuracy for (Strong – Light) using LOOCV.

**Naïve Bayes**		**C4.5**	**Logistic Regression**	**SVM**	**K-Nearest**	**Random Forest**	**Boosting**	**Bagging**
Chest	64.12%	70.39%	68.94%	68.35%	66.00%	**72.35%**	62.25%	70.52%
Wrist	74.24%	79.92%	77.85%	78.17%	78.94%	**83.05%**	79.53%	79.62%
Thigh	60.77%	72.13%	66.55%	64.85%	66.84%	73.78%	63.85%	**76.34%**

**Table 5.** Classification accuracy for (Sudden—Sustained) using LOOCV.

**Naïve Bayes**		**C4.5**	**Logistic Regression**	**SVM**	**K-Nearest**	**Random Forest**	**Boosting**	**Bagging**
Chest	78.15%	82.85%	86.89%	87.46%	85.15%	**87.51%**	84.44%	84.55%
Wrist	78.14%	79.69%	81.66%	82.26%	83.39%	**84.34%**	78.84%	78.25%
Thigh	49.20%	71.87%	66.03%	64.78%	**75.39%**	73.97%	72.45%	73.86%

**Table 6.** Classification accuracy for (Bound—Free) using LOOCV.

**Naïve Bayes**		**C4.5**	**Logistic Regression**	**SVM**	**K-Nearest**	**Random Forest**	**Boosting**	**Bagging**
Chest	80.94%	81.15%	85.13%	85.46%	84.27%	**85.81%**	81.99%	83.19%
Wrist	76.87%	80.63%	87.19%	**87.31%**	82.33%	86.20%	83.04%	84.58%
Thigh	69.73%	72.63%	79.70%	80.05%	75.98%	80.23%	**80.31%**	74.16%

**Table 7.** The accuracy and the F-measure value for the best classifiers for acceleration data obtained from each location.

**Strong—Light**			**Sudden—Sustained**		**Bound—Free**	
Best classifier		Accuracy	Best classifier	Accuracy	Best classifier	Accuracy
		F-measure		F-measure		F-measure
Chest	Random Forest	72.35%	Random Forest	**87.51%**	Random Forest	85.81%
		0.722		**0.875**		0.8559
Wrist	Random Forest	**83.05%**	Random Forest	84.34%	SVM	**87.31%**
		**0.8259**		0.8422		**0.8694**
Thigh	Bagging	76.34%	K-Nearest	75.39%	Boosting	80.31%
		0.7566		0.7462		0.7877

**Table 8.** Best location to place a single accelerometer to detect subcategories within the Effort category.

**Strong—Light**		**Sudden—Sustained**	**Bound—Free**
Best Location	Wrist (Random Forest 83.05%, F-measure = 0.8259)	Chest (Random Forest 87.51%, F-measure = 0.875)	Wrist (SVM 87.31%, F-measure = 0.8694)

**Table 9.** The average accuracy for each location to detect all subcategories within the Effort category.

**Strong—Light**		**Sudden—Sustained**	**Bound—Free**	**Average Accuracy**
Chest	72.35%	87.51%	85.81%	81.89%
Wrist	83.05%	84.34%	87.31%	**84.90%**
Thigh	76.34%	75.39%	80.31%	77.35%
